# Feeding patterns and stunting during early childhood in rural communities of Sidama, South Ethiopia

**DOI:** 10.11604/pamj.2013.14.75.1630

**Published:** 2013-02-26

**Authors:** Masresha Tessema, Tefera Belachew, Getahun Ersino

**Affiliations:** 1 Institute of Nutrition, Food Science and Technology, Department of Applied Human Nutrition, Hawassa University, Hawassa, Ethiopia; 2 College of Public Health and Medical Sciences, Department of Population and Fam. Health, Jimma, University, Jimma, Ethiopia; 3 College of Pharmacy & Nutrition, University of Saskatchewan 110 Science Place, Saskatoon SK CAN S7N 5C9, Canada

**Keywords:** Feeding patterns, early childhood stunting, household, food security, southern Ethiopia

## Abstract

**Introduction:**

The period from birth to two years of age is a “critical window” of opportunity for the promotion of optimal growth, health and behavioral development of children. Poor child feeding patterns combined with household food insecurity can lead to malnutrition which is a major public health problem in developing countries like Ethiopia.

**Methods:**

A community based cross-sectional study that involved 575 participants from rural Sidama was conducted from February to March 2011. A two-stage stratified sampling procedure was employed to select the required households. Multivariable logistic regression analyses were performed to compare stunting by feeding patterns and other characteristics.

**Results:**

Only 14.4% of mothers fed their children optimally. Prevalence of stunting was higher for infants aged 6 to 8 months (43%) than for those in 0-5 months (26.6%) or 9-23 months (39%) category. Women who did not receive antenatal care(ANC) during pregnancy were 1.5 times more likely to practice pre-lacteal feeding and 2.8 and 1.9 times more likely to feed their children below minimum dietary diversity and minimum meal frequency, respectively (*P*=0.01). Mothers older than 18 years during the birth of index child were 86% less likely to feed their child below minimum meal frequency than their younger counterparts (*P*=0.01). Children who started complementary food either before or after the recommended 6 months time, were more likely to be stunted (*P*=0.01).

**Conclusion:**

The feeding practices of most mothers did not meet WHO recommendations. Behavior change communication about the importance of optimal complementary feeding and ANC attendance should be strengthened through extensive use of the Health Extension Workers to reduce the level of child stunting in the study area.

## Introduction

Globally it is estimated that under-nutrition is responsible, directly or indirectly, for at least 35% of deaths in children less than five years of age [1–3]. Under nutrition is also a major cause of disability, preventing surviving children from reaching their full developmental potential [4, 5]. Inappropriate feeding practices may account for approximately one-third of malnutrition, depending on population, place, time and season, and in combination with other causes such as infection and food shortage [6, 7]. In many countries poor breastfeeding and complementary feeding practices are widespread. Complementary foods are often introduced before or after the recommended age of 6 months and are often nutritionally inadequate and unsafe [8, 9]. After a child reaches 2 years of age, it is very difficult to reverse stunting that has occurred earlier unless significant improvement is made in the food security and dietary environment of the child. In the long-term, early nutritional deficits are linked to impairments of intellectual performance; work capacity, reproductive outcomes and overall health during adolescence and adulthood [1, 10–12]. The immediate consequences of poor nutrition during the early formative years include significant morbidity and mortality and delayed mental and motor developments [12].

The 2005 Ethiopian Health Survey showed that 47% of children under five were stunted (< -2 Z-score) and 24% were severely stunted (< -3 Z-score) [13]. While 96% of children (both urban and rural) are breastfed during some period in their lives, it is often not optimal [10]. Nationally, only 69.1% of newborns are put on the breast within 1 hour of birth and less than 80% of infants 2 months old are exclusively breast fed. However, this proportion rapidly drops to 38% at the age of 6 months and complementary feeding starts before 6 months of age in about 14% of infants and after 6 months of age for about 68% [10, 13].

High rates of malnutrition can be attributed to both intrauterine growth retardation and postnatal growth faltering [11, 12]. The postnatal growth faltering in Ethiopia is hypothesized to be largely caused by high rates of infection, limited household (HOUSEHOLD) food availability, and poor infant feeding practices leading to inadequate energy and nutrient intakes [7]. However, studies showing the association of postnatal growth faltering and feeding practices or household food security situation in Ethiopia are limited and are needed to determine the relative contribution of these factors in order to design culturally-appropriate cost-effective, evidence-based interventions. Therefore, the current study aimed to assess feeding practices of children less than two years of age, household food security status and their association with stunting in selected rural communities of Sidama, Southern Ethiopia.

## Methods

### Study setting and Sample

A community based cross-sectional design was employed from February to March 2011 in rural communities of Boricha District. It is located in Sidama Zone, South Ethiopia and 305 km from Addis Ababa. Sidama Zone is the largest Zone in Southern Nations, Nationalities and Regional State (SNNPR) of Ethiopia with an area of 588.15 km^2^. The temperature ranges from (26-33) °C. The district has eight government-funded Health Centers and 38 Health Posts. According to Boricha District Health Office report, the estimated number of infants and young children (aged 0-24 months) from the whole of the District is 16251 (i.e, 6% of the 266,406 total rural population in the district) in the year 2011.

Agriculture is the major source of income for the district. The major crops produced include maize, haricot bean, sweet potato and *enset*, a root crop in the banana family. The community practices both home-gardening (usually growing *enset*, sweet potato, kale and coffee) and farming for other major cereal crops. The district is one of the areas in the region known to experience chronic food insecurity due to the erratic rainfall pattern to which the agriculture is dependent upon. In 2006, an estimated 20.15% of the population was eligible for the productive safety net program, a government program to support food insecure and other vulnerable groups in rural areas [14]. The sample size was determined based on previously published research from the same community which showed a prevalence of stunting ranging from 25% for infants aged 6-8 months to 52% for children aged 12-23 months [15]. Considering the fact that the proportion closer to 50% will give the largest sample size, 52% was used in the sample size calculation using for the formula for estimation of single proportion {n = (Z_1-ε/2_)^2^(1-p)/ ε^2^p} where Z=standard normal variable at 95% confidence level (1.96), p=proportion of stunted children (0.52), ε (epsilon) = relative precision (0.1) [16]. The sample was multiplied by a design effect of 1.5 and 10% was added for non-response giving the final sample size of 585. Three clusters (equivalent to Kebeles, the smallest administrative unit within the district) were selected from a total of 38 by using probability proportional to size method. Then, age stratified lists of children, along with their mothers, were generated from the house-to-house census conducted with the help of the Health Posts in the 3 selected Kebeles. The sampling frames were then used to randomly select approximately equal number of mother-child pairs from the three kebeles. The age strata were used simply to allow comparison of outcome variables since feeding recommendations are different for these age groups [10, 17]. Exact age of each child in months was reported by their mothers and confirmed from immunization cards.

A letter of ethical approval was received from Hawassa University Ethical Clearance Committee. An official letter of co-operation was also obtained from the SNNPR Health Office, Zonal Health Office & the Woreda Health Office. Informed oral consent was secured from study participants in their own language by explaining the purpose of the study, potential risk and benefits of participating in the study and the right to withdraw from the study any time. The participants were also assured about the confidentiality of the data.

### Instrument, measurements and variables collected

A structured questionnaire was used to collect quantitative data on variables pertaining to the socioeconomic and demographic characteristics of participants. The questionnaire was developed in English and translated to Amharic, back-translated to English by independent translator for consistency, and then pre-tested. The mother of the index child was interviewed to provide answer to questions other than child anthropometry. All the interviews, measurements and testing were conducted at the residences of the study participants. The data were collected by interviewers who had completed 12^th^ grade and who took an intensive training for two days on the content of the questionnaire and on general approaches to data collection.

Feeding practice related variables were assessed using eight core and optional feeding practice indicators developed by the World Health Organization (WHO) for assessing the adequacy of infant and young child feeding (IYCF) practices. These indicators include early initiation of breastfeeding (i.e., within one hour after birth), exclusive breastfeeding (EBF) under 6 months, continued breastfeeding at 1 year, introduction of solid, semi-solid or soft foods, minimum dietary diversity, minimum meal frequency, minimum acceptable diet, and bottle feeding [17]. WHO also defines optimal infant feeding as initiating breastfeeding within one hour of birth, breastfeeding exclusively for the first six months, starting complementary food at six months post-delivery, continuing to breastfeed for two years, breast feeding day and night at least 8 times, the giving of colostrum, no pre-lacteal feeds, no bottle feeding and responsive feeding of solid, semi-solid food [17]. In our study sub-optimal feeding practice was defined as lack of compliance to any of these recommended practices. Birth order of the index child was assessed to investigate its association with EBF practice under 6 months as recommended.

Stunting, linear growth deficit for a specific age and sex was used as outcome variable and we investigated its association with IYCF practices and other socioeconomic and demographic profiles, HOUSEHOLD food security and maternal practices. In addition, sub-optimal practices [specifically giving of pre-lacteal feed (giving of liquids or foods other than breast milk prior to the establishment of regular breast-feeding) feeding below minimum meal frequency and below minimum diet diversity] were also fitted as separate outcome variables in multivariate logistic model and their associations with other explanatory variables were examined.

Mothers were also asked if they attended antenatal clinics (ANC), received advice on complementary feeding and consumed additional food (more meal and /or snacks) during pregnancy and/or lactation of the index child. These questions were asked to investigate if mothers were aware of the increased nutrient requirement during these physiological stages and also to examine their association with the outcome variables.

Four levels (Food secured, mild, moderate, and severely food insecure) of household food security status were assessed based on the Food and Nutrition Technical Assistance (FANTA) guideline [18]. Bivariate analyses were carried out to identify the potential association of household food security and stunting.

The principal investigator performed all anthropometric measurements with assistants on the infants and young children to eliminate inter-examiner error, using standard procedure as outlined in Gibson (2005) [19]. Measurements taken include length, head circumference and weight. To measure length was measured while child without shoes was kept in supine position with knees kept straight and feet positioned at 90° to the legs. Both length and head circumference were measured to the nearest 0.1cm. Weight of the lightly clothed infants and children were measured to the nearest 10g. Weight was measured using an electronic hanging scale (Seca, Hanover Germany). All instruments were calibrated before measurements took place.

Dietary diversity score (the number of food groups the child consumed during the 24-hours preceding the survey) was used as a proxy for quality of diet consumed. It was calculated and divided into two categories of meeting the minimum Dietary diversity or not (i.e., consumption of < 4 and ≥ 4 food groups) based on the WHO guidelines [17]. Households were also asked if they grew vegetables so as to analyze its effect on the dietary diversity of foods given to children.

### Data analysis

All continuous data were checked for normality using the Kolmogorov-Smirnov test. Study subjects were grouped into three age groups: 0-5 months, 6-8 months, and 9-23 months. Stunting was assessed using the anthropometric index length-for-age Z-score (LAZ) by applying the WHO 2006 multi center growth reference data using the computer program WHO Anthro 2007 (version 3.2.2). A z-score < 2 standard deviations (SD) below the median value of the reference population was used to define stunting. Similarly, weight-for-height < -2 SD was used to define wasting.

First the raw data were entered, cleaned and analyzed using SPSS statistical software (SPSS Inc. version 17.0, Chicago, Illinois). Descriptive statistics (frequencies, percentage, mean, ± SD) used to present counts, proportions and averages. Binary logistic regression models were applied to select variables that are candidate for multivariable model. The variables that were significantly associated with stunting on the bivariate analysis were used in the multivariable regression model to identify their independent effect. We present the anthropometric results as proportions, and the output of the logistic regression as Adjusted Odds Ratios (AOR) with 95% confidence intervals (CI). Associations were reported as significant at p-values < 0.05.

## Results

The final analysis included 575 mother-infant pairs for which a complete data were obtained making the response rate 98.5%. From the infants and young children (IYC) included in the study, 286 were males and the remaining 289 were females aged 0-23 months. The median age of the children was 12 months. The mean (±SD) family size of the study participants was 5.4 (±1.9) persons. Nearly two thirds (62%) of the households had 5 or more family members. More than half of households (54%) had two or more children less than 5 years of age. A majority (96%) of the children studied lived in male-headed households. The prevalence of parents with no formal education was higher (72.3%) for mothers than fathers (53.6%). More than half of the mothers (64%) had attended antenatal clinics during their pregnancy. Almost all mothers (99.8%) reported vaginal delivery at their own home (97.6%).

Regarding consumption of extra food during pregnancy and/or lactation of the index child, only 33.4% of mothers reported consumption of additional foods than when they were not pregnant or lactating.

The result of foods and drinks consumed by IYC in the 24 hours preceding the survey showed that the median diet diversity score for the study participants was two. Eighty-six percent of the children had dietary diversity below the minimum dietary diversity recommended by the WHO (< 4 food groups). Proportion of households that reported growing of any vegetable was 102.7%. The majority (93.1%) of mothers in this study reported that IYC consumed complementary foods made from grains, roots, and tubers (specifically corn bread and potato). Most of the children (62%) did not consume any fruits and vegetables during the preceding 24 hours before the survey. Moreover, only 6.3% of children consumed vitamin-A rich fruits and vegetables such as carrot or yellow pumpkin in the 24-hours preceding the survey. In this study, only 1.9% of the infants and young children consumed meat, fish, or poultry and 3.4% consumed eggs in the 24 hours before the survey.

The result from food frequency questionnaire (data not shown) showed that consumption of beef, fish, chicken was not frequent. Children consumed these flesh foods once per month or less throughout the year; whereas cereal-based food, mainly maize, was consumed daily by most of study subjects. Daily consumption of root and tuber, mainly sweet potatoes and potatoes, were reported only for about 2.3% of the children. Fruit and vegetable consumption by children was very minimal (once or twice per week or less) except for very few families (≤ 2.4%) who reported daily consumption of kale, avocado and banana.

The assessment of food security status of households revealed that 21.2% were food secured whereas 6.4%, 34% and 38.4% were mildly, moderately and severely food insecure, respectively.

The prevalence of stunting was higher (43%) for children aged 6-8 months than those 0 to 5 and 9 to 23 months, whereas the corresponding wasting was higher (9.6%) for 9 to 23 months old children than those 0-5 and 6 to 8 months of age (Table 1). The highest proportion of underweight (Weight-for-age < -2SD) was for children in the category of 9 to 23 months (29%).

**Table 1:** Mean ±SD anthropometric measurements of infant and young children, Boricha District, South Ethiopia, 2011

Anthropometric measurements*	Age in months (n=575)							
	Age 0-5 months (n=109)		Age 6-8 months (n=79)		Age 9-23 Months (n=387)		Age0-23 months (n=575)	
Length(cm)	56.7±4.6		63.6±3.4		74.8±6.4		69.8±9.4	
Weight(kg))	5.02±1.2		6.7±1.1		8.8±1.5		7.8±2.1	
Head circumference(cm)	39.5±2.7		43±1.8		46.5±2.04		44.7±3.5	
Length-for-age z-score)	-1.1±1.3		-1.9±1.3		-1.6±1.7		-1.5±1.6	
Weight-for-age z-score)	-1.0±1.2		-1.5±1.3		-1.29±1.27		-1.3±1.2	
Weight-for-height z-score)	0.08±1.5		-0.3±1.2		-0.6±1.2		0.51±1.2	
Head circumference-for-age z-score	0.2±1.4		-0.2±1.2		0.2±1.4		0.15±1.4	
Nutritional status	**n**	**%**	**n**	**%**	**n**	**%**	**N**	**%**
Prevalence of stunting (%)	29	26.6	34	43.0	151	39	214	37.2
Prevalence of wasting (%)	7	6.4	4	5.1	37	9.6	48	8.3
Prevalence of underweight (%)	26	23.9	23	29.1	98	25.3	147	25.6

* Values are mean ± SD

The majority (93.6%) of the mothers reported initiating breast-feeding their children within 1 hour after birth. The overall rate of EBF for infants under 6 months was 62.4%; however, EBF rates declined from 67.7% in the first month to 60.6% for age 4-5 months. Bottle feeding (specifically bottles with nipples at their tips) is not recommended because improper sanitation associated with bottle-feeding can introduce pathogens to the infant. The present study found that total prevalence of bottle-feeding was 7.5% (2.8%, 7.6% and 8.8% for age groups 0-5, 6-8 and 9-23 months, respectively). Prevalence for timely introduction of complementary foods for 6-8 months old children was 57(72.2%). Compared to the WHO recommendations for IYCF practices, only few (14.4%) of the children were fed with complementary diet of minimum dietary diversity (≥4).

To meet energy requirements WHO [17] also recommends a minimum of two and three meals per day for breastfeeding IYC between the age 6-8 and 9-23 months, respectively. With reference to these recommendations, the results in the current study showed that 30(38%) of IYC in the age 6-8 months and 147(37%) in the age 9-23 months were reported to receive below the recommended minimum meal frequency. In addition, proportion of IYC who were fed with minimum acceptable diet (an indicator that combines minimum meal frequency and minimum diet diversity) were 4(5.1%) for those in the age group 6-8 months and, 42(10.9%) for those 9-24 months of age.

Based on the WHO IYCF practices indicators, majority (85.6%) of the mothers sub-optimally fed their children, i.e, they did not follow at least one or more of the recommended practices for optimal feeding for their children from 0-24 months of age. And also 40.6% of the mothers reported practicing pre-lacteal feeding.

On multivariable logistic regression after adjustment for other explanatory variables, pre-lacteal feeding was positively associated with child not fed with minimum meal frequency per day, mother who did not receive advice about the complementary feeding and did not follow antenatal care (ANC) during pregnancy. Maternal age during the first child birth > 18 years and mother who breastfed less than eight times per day were negatively associated with pre-lacteal fed (Table 2). The birth order of index child was also find to significantly predict EBF practice. Mothers were 5.5 times more likely to practice EBF for their first child than for a child 4^th^ and above birth order (AOR=5.5; 95% CI: 1.09 - 27.72, p< 0.05). The study showed that mothers who did not receive advice about complementary feeding were 2.3 times more likely to practice pre-lacteal feeding (AOR=2.3; 95%CI: 1.29 - 4.16]) than those who did receive information. Mothers who fed their child below minimum meal frequency were twice more likely to practice pre-lacteal fed than their counterpart (AOR= 2.01; 95% CI:1.29 - 3.24); moreover mothers who did not follow ANC during pregnancy were 1.5 times more likely to practice pre-lacteal feeding than those who did follow ANC (AOR=1.5; 95% CI: 0.93 - 2.47). The study also showed that mothers whose age was greater than 18 year during the first child birth were 55% less likely to practice pre-lacteal feeding than their younger counterparts (AOR= 0.45; 95% CI: 0.29 - 0.70). Mothers who breastfed less than eight times per day were 80% less likely to practice pre-lacteal feeding than those who fed more times (AOR=0.2 [95%CI: 0.10,0.39).

**Table 2:** A bivariate and multivariate logistic regression output showing the odds of giving Pre-lacteal feeding for infant and young children in Boricha District, South Ethiopia, 2011

	Pre-lacteal fed(n=575)			
Variables	Yes No. (%)	No No. (%)	Crude OR (95%CI)	Adjusted OR(95%CI)
Maternal age during first birth				
15-18	77(13.8)	154(26.7)	1	1
>18	156(27)	188(32.6)	0.60(0.43,0.85)**	0.45(0.29,0.70) **
Delivery assisted				
TBA	50(8.7)	79(13.7)	1	1
HEW	2(0.33)	9(1.6)	2.9(0.59,13.73)	6.2(0.62,62.57)
Nurse	1(0.17)	8(1.4)	5.1(0.62,41.71)	3.9(0.30,51.49)
Parents	180(31.3)	246(42.8)	0.87(0.58,1.29)	0.8(0.46,1.34)
Follow ANC				
Yes	161(28)	207(36)	1	1
No	72(12.5)	135(23.5)	1.5(1.02,2.08) *	1.5(0.93,2.47) *
Receive advice on complementary feeding				
Yes	147(25.6)	176(30.6)	1	1
No	86(14.9)	166(28.9)	1.6(1.15,2.27) **	2.3(1.29,4.16) **
Minimum Meal frequency				
Yes	144(25)	153(26.6)	1	1
No	89(15.5)	189(32.9)	2(1.42,2.81)**	2.01(1.29,3.24)**
Frequency of Breast feeding/day				
> 8 times	182(31.9)	313(54.8)	1	1
< 8 times	51(8.9)	25(4.4)	0.29(0.17,0.48)*	0.2 (0.10,0.39)*

* Significant at <0.05

** Significant at <0.01 OR= Odds Ratio; TBA = Traditional Birth Attendance; HEW= Health Extension Workers; ANC= Antenatal Clinic

Children whose fathers had gotten no formal education were 2.9 times more likely to go through early introduction of complementary food than their counter part ((AOR=2.9; 95% CI: 1.3, 6.5). Moreover, mother who practices bottle feeding were 3.1 times more likely to feed their child early complementary food than their counter parts (AOR=2.9; 95%CI: 1.3, 6.5). Whereas mothers that fed their child pre-lacteal feed were 4.5 times more likely to conduct early introduction of complementary food than their counter parts (AOR=4.5; 95%CI: 2, 9.6).

Households whose land size was less than 0.25 hectare, fathers with no formal education, mothers who reported no increased food consumption during lactation and pregnancy were positively associated with late introduction of complementary food. The finding showed that with land size less than 0.25 hectare were 2 times more likely to practice late introduction of complementary food than their counter parts (AOR=2; 95%CI: 1.1 - 3.3). Households with fathers having no formal education were 2 times more likely to practice late introduction of complementary food than those having formal education (AOR=2; 95%CI: 1.3 - 3.4). Whereas mother that did e not practice the consumption of extra food during pregnancy and lactation were 2 times more likely to start late complementary food than their counter part respectively.

Approximately 86% of mothers in the study areas fed their child below the minimum dietary diversity recommended by WHO (recommended=4, study mean= 2). After adjustment for explanatory variables by logistic regression, HOUSEHOLDs that did not grow vegetables, mother who did not follow ANC and mother who did not consume extra meal during pregnancy or lactation were positively associated with child fed below minimum dietary diversity score. HOUSEHOLD land size greater than 0.25 hectare and later birth order were negatively associated with child fed below minimum dietary score (Table 3).

**Table 3:** A bivariate and multivariate logistic regression output showing the odds of being “fed below the minimum dietary diversity” for infant and young children in Boricha District, South Ethiopia, 2011

Variables	Yes No. (%)	No No. (%)	Crude OR(95%CI)	Adjusted OR(95%CI)
Grow Vegetables				
Yes	20(4.3)	64(13.7)	1	1
No	47(10.1)	335(71.9)	2.2(1.25,3.93)**	2.8(1.33,6.06)**
ANC follow up				
Yes	48(10.3)	258(55.4)	1	1
No	19(4.1)	141(30.3)	1.5(0.85,2.60)	2.8(1.25,6.14)*
Feeding style				
infant is usually encouraged to eat more	38(8.4)	269(60)	1	1
infant is usually not encouraged to eat more	25(5.6)	94(21)	1.5(0.48,4.62)	0.4(0.19,0.85)*
infant is usually forced to eat more	4(0.9)	18(4)	0.82(0.26,2.62)	0.40(0.11,2.04)
Consume extra food during lactation pregnancy				
Yes	28(6)	133(28.5)	1	1
No	39(8.4)	266(57.1)	1.5(0.89,2.53)	2.6(1.30,5.35)**
Land size				
< 0.25 hectare	12(2.6)	150(31.2)	1	1
≥ 0.25 hectare	55(11.8)	248(53.4)	0.39(0.20,0.74)**	0.26(0.11,0.66)**
Birth order				
First	7(1.5)	83(17.8)	1	1
Second	14(3)	100(21.5)	0.61(0.24,1.58)	0.16(0.04,0.68)*
Third	12(2.6)	61(13.1)	0.40(0.18,1.28)	0.14(0.03,0.68)*
Fourth and above	34(7.3)	155(33.3)	0.40(0.17,0.90)*	0.31(0.15,0.66)**

* Significant at <0.05

** Significant at <0.01

It was found that those households that did not grow vegetables were 2.8 times more likely to feed their child below minimum dietary diversity than their counterparts (AOR=2.8; 95%CI: 1.33 - 6.06). Whereas mothers who did not follow ANC during pregnancy were 2.8 times more likely to fed their child below minimum dietary diversity than those who follow ANC (AOR=2.8; 95%CI: 1.25 - 6.14). The study also showed that mother who did not consume extra food during lactation/pregnancy were 2.6 times more likely to feed their child with low minimum dietary diversity than their counterpart (AOR=2.6; 95%CI: 1.30 - 5.35). On the other hand, children in households with land size > 0.25 hectare and with 4^th^ or above birth order were 73% and 69% less likely to be fed below minimum dietary diversity than their counterparts (AOR=0.265; 95%CI: 0.11 - 0.66) and AOR=0.31; 95%CI: 0.15 - 0.66 respectively) (Table 3).

Mothers who did not follow ANC were 1.9 times more likely to feed their child below minimum meal frequency than their counter parts (AOR=1.9; 95% CI: 1.14 - 3.04). Mothers who forced their children to eat more than they take themselves during complementary feeding were 4.2 times more likely to fed below minimum meal frequency than those who simply encouraged the child to eat more (AOR=4.2; 95% CI:1.5 - 11.89). This study also found that children with birth interval less than two years were 2.7 times more likely to be feed below minimum meal frequency than those who were the first born (AOR=2.7; 95%CI:1.15 - 6.17). Mothers > 18 years of age during index child birth were 86% less likely to feed their children with lower meal frequency than their counterparts (AOR=0.14; 95% CI: 0.03 - 0.62) (Table 4).

**Table 4:** A bivariate and multivariate logistic regression output showing the odds of being fed below the minimum meal frequency for infant and young children in Boricha District, South Ethiopia, 2011

Variables	Yes	No	Crude OR(95%CI)	Adjusted OR(95%CI)
	No. (%)	No. (%)		
Delivery assisted				
TBA	78(16.7)	25(5.4)	1	1
HEW	7(1.5)	1(0.2)	0.45(0.05,3.80)	0.54(0.05,6.43)
Nurse	4(0.9)	2(0.4)	1.5(0.27,9.03)	1.4(0.22,9.39)
Relatives	208(44.6)	142(30.5)	2.1(1.29,3.48)**	2.2(1.17,3.96)*
ANC follow				
Yes	208(44.6)	98(21)	1	1
No	89(19.1)	71(15.2)	1.7(1.14,2.51)**	1.9(1.14,3.04)*
Feeding style				
usually encouraged to eat more	202(43.4)	105(22.5)	1	1
usually not encouraged to eat more	85(18.2)	34(7.3)	0.8(0.48,1.22)	0.82(0.49,1.39)
usually forced to eat more	8(1.7)	14(3)	3.4(1.37,8.28)**	4.2(1.5,11.89)**
Mother take Vitamin A supplementation				
Yes	100(21.5)	80(17.2)	1	1
No	197(42.3)	89(19.1)	0.57(0.38,0.83)**	0.47(0.29,0.76)**
Birth interval between index child				
First birth	42(9)	21(4.5)	1	1
Less than 2 years	98(21)	70(15)	1.4(0.78,2.62)	2.7(1.15,6.17)*
Greater than or equal 2 years	157(33.7)	78(16.7)	0.9(0.55,1.79)	2.1(0.91,4.77)
Household food security status				
Food Secure	49(10.5)	47(10.1)	1	1
Mildly Food Insecure	20(4.3)	9(1.9)	0.47(0.19,1.13)	0.27(0.09,0.82)*
Moderately Food Insecure	111(23.8)	55(11.8)	0.51(0.30,0.86)*	0.46(0.25,0.87)*
Severely Food Insecure	117(25.1)	58(21.5)	0.52(0.03,0.86)*	0.40(0.21,0.77)**
Mother age during index child birth				
15-18years	4(0.8)	8(1.7)	1	1
> 18years	293(63)	161(34.5)	0.28(0.08,0.93)*	0.14(0.03,0.62)*

* Significant at <0.05

** Significant at <0.01

Table 5 presents logistic regression output on the predictors of stunting. The analysis showed that children of mothers who did not increase food consumption during pregnancy and lactation were 1.6 more likely to be stunted than their counter parts (AOR=1.6; 95%CI: 1.06 - 2.3). The study also revealed that time of complementary food was associated with stunting. Children of mother who had started complementary feeding before six months were 3.2 times more likely to be stunted than their counterparts (AOR=3.2; 95%CI: 1.6 - 6.6). This study also indicated that children who experienced late introduction of complementary food (after 6 months) were 2.3 times more likely to be stunted than their counter parts (AOR=2.3; 95% CI: 1.3, 4.05). The rest of the observed feeding patterns were not significantly associated with stunting.

**Table 5:** Bivariate and multivariate logistic regression outputs showing the odds of being stunted for infants and young children (aged 0-23 months) by various feeding pattern characteristics in Boricha District, South Ethiopia, 2011

Variables	Stunted(n=575)			
	Yes	No	Crude OR(95%CI)	Adjusted OR(95%CI)
	No. (%)	No. (%)		
Time complementary food started				
Less than six months	21(3.7)	22(3.8)	2.9(1.41, 5.79)*	3.2(1.6, 6.6)**
greater than six months	43(7.5)	48(8.3)	2.3(1.31, 4.15)*	2.3(1.3, 4.05)**
At six month	117(20.3)	201(35)	1.6(1.003, 2.51)*	1.6(1.02,2.6)
Not started	33(5.7)	90(15.7)	1	1
Frequency of BF within 24 hours (n=571)				
> 8 times	33(5.8)	43(7.5)	1	
< 8 times	180(31.5)	315(55.2)	0.75(0.46,1.21)	
Consumption of extra food during pregnancy/lactation				
Yes	60(10.4)	132(23)	1	1
No	154(26.8)	229(39.8)	1.5(1.03,2.14)*	1.6(1.06,2.3)*
Feeding style (n=455)				
usually encouraged to eat more	122(26.9)	185(40.9)	1	-
usually not encouraged to eat more	52(11.5)	70(15.5)	1.3(0.43,4.01)	
usually forced to eat more	7(1.6)	16(3.6)	0.53(0.05,5.3)	
Pre-lacteal feeding				
Yes	92(16)	141(24.5)	1.2(0.84,1.66)	
No	122(21.2)	220(38.3)	1	
Bottle feeding				
Yes	19(3.3)	24(4.2)	1	
No	195(33.9)	337(58.6)	0.73(0.39,1.37)	
Minimum Meal Frequency/day (n=466)				
Yes	114 (24.5)	183(39.3)	1.2(0.79,1.71)	
No	71 (15.2)	98(21)	1	
Minimum Dietary Diversity (n=466)				
Yes	28 (6)	39(8.4)	0.9(0.53,1.53)	
No	157(33.7)	242(51.9)	1	
Minimum acceptable diet (n=466)				
Yes	20(4.3)	27(5.8)	0.88(0.48,1.62)	
No	165(35.4)	254(54.5)	1	

* Significant at <0.05

** Significant at <0.01, OR = Odds Ratio; CI: confidence interval; BF = Breast feeding

## Discussion

Infant and young child feeding (IYCF) patterns constitute a major component of child caring practices. This study showed that stunting was significantly associated with IYCF practices that include late and early introduction complementary foods. Both early and late introduction of liquids and solid food reduces the duration and frequency of breastfeeding and increases risk of infant morbidity and mortality; therefore such unhealthy behavior needs to be discouraged.

A study in Bangladesh that considered IYCF practices and anthropometric outcomes among children 0-23 months of age supports our findings. The study reported significant positive associations between EBF practices and higher weight-for-height z-score, between timely start of complementary feeding and higher height-for-age z-scores, and also reported a protective effect of higher diet diversity score on stunting and underweight [20]. The current study showed children who started complementary foods late were 2.3 times more likely to be stunted than their counterparts. In addition, our study found that children who stared complementary foods before 6 months were 3.2 times more likely to be stunted than their counter part. We propose this effect may be the negative impact of complementary feeding on breastfeeding frequency and duration. However, a study conducted in northern Senegal contradicted our findings, as these researchers reported no significant association between wasting or stunting and early introduction of complementary food [21]. A study in North Ethiopia (West Gojam Zone) also showed that, among other factors, early or late introduction of complementary foods, pre-lacteal feeding, food quality and duration of breastfeeding had a highly significant associations with under-five stunting [22].

The finding in our study showed that mothers who did not increase consumption during pregnancy and lactation were 1.6 times more likely to have a stunted child than those who did increased consumption. This is in-line with the fact that poor maternal dietary practices are related with child nutritional status. A study conducted in Burkina Faso stated that dietary diversity scores and frequency of meals were positively associated with stunting [23]. Arimond and Ruel, using evidence from a meta-analysis of 11 demographic and health surveys, have also reported positive association between child dietary diversity and nutritional status that is independent of socioeconomic factors [24]. However our data did not show any association between dietary diversity or minimum meal frequency and stunting.

Household food insecurity causes hunger and malnutrition in most countries in the world [25]. Food insecurity is at emergency levels in Ethiopia for around 5 million people [25, 26], leading to high level of childhood stunting. Although food insecurity and malnutrition have become policy priorities in Ethiopia, the factors mediating the relationship between these two challenges needs to be elucidated so they can be specifically targeted to reduce mortality and morbidity. In this study, household food security status was not associated with stunting despite the high prevalence of food insecurity reported. This finding is consistent with the reports of similar studies done in Nepal Kailali District and Colombia where household food insecurity did not associate with stunting even though high prevalence of food insecurity were reported [27, 28]. However, follow up studies in conducted in rural Bangladesh contradicted this finding as they found a significant association between HOUSEHOLD food insecurity and appropriate IYCF practices with childhood stunting [29, 30].

There is no significant difference in the mean length-for-age among the three groups. The result showed that the overall prevalence of stunting rate in the first two year was 37.2%, which is slightly higher than the 2005 Ethiopian Demographic Health Survey (EDHS) report value (35.1%) [13]. Stunting was more prevalent among children 6-8 months old (43.0%), which was almost two times that observed among children aged 0-5 months (26.6%), but was lower than the prevalence for those aged 9-23 months (39%). The observed prevalence was still higher for the first two age groups (0-5 and 6-8 months) when compared to the national figure for the same age group in the 2005 EDHS. The prevalence of stunting, reported by an earlier study in the district, ranged from 25% for infants aged 6-8 months to 52% for children aged 12-23 months [15]. The higher prevalence of stunting reported for the infants aged 6-8 months in the current study supports the fact that this period (6-8 months) is transition time for starting complementary food because breast milk alone is no longer sufficient to meet energy and nutrient requirements of the child. Thus, this period might put some stress in terms of meeting requirements until the child gradually adjusts to the complementary food. This might also indicate that children in the age group 6-8 months were at higher risk of being stunted compared to other age group. WHO defines optimal infant feeding as initiating breastfeeding within one hour of birth, breastfeeding exclusively for the first six months, starting complementary food at six months post-delivery, continuing to breastfeed for two years, breast feeding day and night at least 8 times, the giving of colostrum, no pre-lacteal feeds, no bottle feeding and responsive feeding of solid, semi-solid food [17]. Depending on this composite definition, majority of mothers were not properly feeding their child. The prevalence of optimal feeding practices in these communities was only 14.4% which is lower than 22% reported in Ethiopia by IYCF guidelines [13].

The practice of pre-lacteal feeding is reported in many developing countries [17, 22, 31] and is a practice that is not recommended by the WHO because it increases the risk of gastrointestinal infection, deprives the child of colostrum, and discourages EBF practices and the benefits associated thereof. This study found that pre-lacteal feeding is a common, deep-rooted tradition for the first 2 to 3 days. Pre-lacteal feeding was given to about 41% of infant in this study; the common pre-lacteal food was Amesa (herbs mixed with water). This finding was higher than the 2005 EDHS report where 29% at national level and 15% in SNNPR were given a pre-lacteal feed. The finding suggests pre-lacteal feeding is still prevailing in the area despite the nutrition education program currently running under the national Health Extension Program in those communities. A more targeted effort in educating the communities about the danger of pre-lacteal feed upon the health of newborns may alleviate the problem. However, these findings were lower when compared to a study report in Nairobi-Kenya where 51.3% infants were given pre-lacteal feeds, while even higher proportion (65%) of infants reported receiving pre-lacteal feeds in Kanartaka- India [32, 33]. The Study conducted in Ethiopia West Gojam Zone, also showed pre-lacteal feeding was one of the feeding practices associated with stunting, but not in present study [22].

Older maternal age during first child birth was found protective. Those mothers whose age during first child birth were greater than 18 years were 55% less likely to practice pre-lacteal feeding than those ≤18-year. This may be related with better knowledge by older mothers. ANC follow up during pregnancy and meal frequency were important predictors for pre-lacteal feeding practices. The study reveals that those mothers who did not follow ANC were 1.5 time more likely to practice pre-lacteal feeding than those who did. This may be due to the fact those mothers who follow ANC get nutrition education from the health workers. In a similar study conducted in India, mothers receiving nutritional education were more likely not to use pre-lacteal feed [33]. Mothers who fed their child below minimum meal frequency per day were 2 times more likely to practice pre-lacteal fed than those who fed with minimum meal frequency per day. The reason for this could be that mothers who frequently fed their child have better nutrition knowledge.

Bottle feeding across the different age groups (2.8% in 34). Thus the 2.8% prevalence of bottle feeding in infants less than six months in our study shows bottle feeding is not a huge problem for now. However, we didn't investigate if this was the result increased awareness of the risk of bottle feeding or just because the mothers didn't afford to. In this study the practice of EBF for infants under 6 months of age (according to the WHO, guidelines) was 62.4%, as opposed to 49% reported by EDHS 2005 [13]. Generally EBF decreases as infants aged. At the end of the first month of life, the rate of EBF was 67.7%. Later the rate dropped to 60.6% by age 4-5 months. This was still twice as high as the national figure (31.6%) reported by the 2005 EDHS for age 4-5 months. Similar study was conducted in South Gujarat region of India which reported a higher EBF prevalence rate than the national estimate [35]. Future studies may investigate whether reported practice of EBF in the study area was the result of the health extension program or some other causes.

We found that birth order is a significant predictor of excusive breast feeding. A first child was 5.5 more likely to receive exclusive breast feeding than a child whose birth order was fourth and above. The probable reason for this might be that when mothers have more children, the time they may spend caring for the youngest child is less or the baby may spend more time with older children that there is less time to breastfeed. In present study, 23.6% of mothers introduced complementary food early and late. Finding of the present study also states that paternal education was significantly associated with early and late introduction of complementary foods. Those who had no formal education were 2.9 times more likely to practice early introduction of complementary food than their counterparts and children of fathers having no formal education were also 2 times more likely to start complementary food late than their counterparts. Land ownership is considered as a wealth index in developing countries [24]. In present study household with land size size <0.25 hectare were two times more likely to practice late introduction of complementary foods.

Diets that are diversified reflect higher dietary quality and greater possibility of meeting daily energy and nutrient requirements [17, 36]. Ensuring minimum dietary diversity is particularly critical for vulnerable children because they need energy and nutrient dense foods to grow and develop both physically and mentally and to live a healthy life [24]. The study showed that 86% of mothers in the study fed their child below the minimum dietary diversity recommended by WHO. Households that did not grow vegetables were 2.8 times more likely to feed their child below minimum dietary diversity than their counterparts. Moreover, mothers that did not receive ANC were 2.8 times more likely to feed their child below minimum dietary diversity than those who received ANC. This might be due to the lack of knowledge about proper IYCF practices offered during ANC visits by Health Extension Workers to which those who attended ANC stand in advantage. Similar studies conducted in Bangladesh support this finding [36].

A Study showed that eating more frequently per day improves a child's nutritional status as well as its ability to achieve and maintain healthy growth [37]. It was found that lower minimum meal frequency was significantly associated with a lack of ANC follow up and birth interval. Mothers who did not receive ANC were 1.9 times more likely to feed below minimum meal frequency than those who received ANC. Moreover, children with birth interval of less than two years were 2.7 times more likely to feed below minimum meal frequency than those whose birth was first. This might be due to the combined effect of not getting ANC counseling and having closely spaced young children whose need the mother may fail to meet.

## Conclusion

In conclusion our finding showed that prevalence of optimal child feeding practices recommended by the WHO was very low (14.4%). Birth order, receiving nutritional advice during pregnancy, ANC attendance and maternal age ≤ 18 years were important predictors of sub-optimal child feeding practices. Prevalence of stunting was higher in children aged 6-8 months compared to other age groups implying the consequences of suboptimal complementary feeding on child growth. Along with maternal dietary practices during pregnancy and lactation, the practice of both early and late introduction of complementary foods (i.e., not at 6 months) was significantly associated with stunting.

The following are some of the strengths of this study: it was a community-based assessment with almost 99% response rate and aimed at assessing mothers real IYCF experiences while staying in their own natural environment. It also formulates a test hypothesis to determine whether suboptimal feeding practices and household food security status were associated with early childhood stunting in the study community. Lager sample size was used relative to the national survey. However, since the study was observational study, causal associations between IYCF practices and other socioeconomic determinants with stunting at early age cannot be established. We didn't also assess real reasons “why” some practices (e.g. prevalence of EBF, low rate of bottle feeding) were better in those communities relative to the national figures. Future studies may integrate the collection of some qualitative data to complement the “why” issues. A follow-up study may also help to establish causal associations. We recommend strengthening the nutrition component of current health extension programs in the study area to specifically target the promotion of IYCF practices as recommended by WHO and the national guideline.
